# Editorial: Cerebral microdialysis

**DOI:** 10.3389/fneur.2023.1266540

**Published:** 2023-08-07

**Authors:** Jefferson W. Chen, Alex B. Valadka, M. Ross Bullock, Keri L. H. Carpenter

**Affiliations:** ^1^Department of Neurological Surgery, University of California, Irvine, Orange, CA, United States; ^2^Department of Neurological Surgery, University of Texas Southwestern Medical Center, Dallas, TX, United States; ^3^Department of Neurological Surgery, University of Miami, Coral Gables, FL, United States; ^4^Division of Neurosurgery, Department of Clinical Neurosciences, University of Cambridge, Cambridge, United Kingdom

**Keywords:** acute brain injury, cerebral microdialysis, traumatic brain injury, glymphatics, dural lymphatics, extracellular fluid

Cerebral microdialysis was pioneered in 1974 by Professor Urban Ungerstedt in animal models to assess neurotransmitters in the extracellular space (1). This technique has been used clinically since 1995, largely for the analysis of cerebral metabolites via bedside enzymatic analysis of glucose, pyruvate, lactate, glutamate, and glycerol (Nordstrom et al.). Numerous studies in patients with severe traumatic brain injury (TBI) and stroke have provided information about the changes that occur in the brain after such insults. Several robust relationships that are reflective of the status of the injury can be seen between the interstitial levels of glucose, lactate, and pyruvate (2–6).

When we embarked upon this Research Topic, our mission was to invite members of the scientific community to publish their work involving cerebral microdialysis with the hope of bringing to light many of the most recent findings. The members of our editorial board are all very experienced with personally conducting cerebral microdialysis at their respective institutions, with a cumulative experience of about 80 years.

We were very privileged to receive scholarly work from some of the pioneers in cerebral microdialysis who continue to be innovators in this field (Nordstrom et al.; Sharma et al.; Stovell et al.). Nordstrom et al. describe the interesting use of microdialysis to compare the metabolites in jugular venous bulb blood with those in the brain. They report very good correlates for global cerebral ischemia after aneurysmal subarachnoid hemorrhage (Nordstrom et al.). They also review the important tenets and precautions regarding cerebral microdialysis (CMD) that they have learned over many years. Key to the acquisition and interpretation of results from CMD is the location of the catheters within the injured brain. This important principle is highlighted in this article as well as several others in this Research Topic (Falcone and Chen; Nordstrom et al.; Stovell et al.).

Stovell et al. point out that there are just 30 centers in Europe and the United States that are routinely using cerebral microdialysis in clinical care. This is a small number given the large number of patients that suffer from acute brain injury. We have placed microdialysis catheters for our own studies and are very cognizant of the challenges in getting the catheter in the “right place,” given that microdialysis is a focal technique. We prefer to sample the zone of “at-risk” brain in order to optimize the brain environment for that region, as well to monitor a region more representative of the overall brain (7, 8). Often, a microdialysis catheter is placed as part of multimodality monitoring, inserted via a right frontal cranial access device into radiologically normal-appearing brain (7). Techniques to optimize placement and maintain a robust sampling system for monitoring in the ICU over the subsequent days are described in detail (Falcone and Chen, Stovell et al.). These methods to ensure good placement and a steady stream of reliable data serve as a useful guide for any centers that are contemplating the establishment of a CMD program. The relationships between glucose, lactate, pyruvate, and the lactate/pyruvate ratio have been examined for many years, and the findings for ischemia, metabolic stress, and mitochondrial dysfunction have been nicely worked out. Translation of these changes to improve outcome is the challenge with CMD and other brain monitoring modalities (Sharma et al.).

In the article by Sharma et al. their figure 1 reminds us of the ability of CMD to sample the extracellular space in the brain. In addition to examining the effects of therapies on brain metabolites in TBI or acute brain injury (i.e. stroke, aneurysmal subarachnoid hemorrhage), CMD has been used to examine the levels of chemotherapeutic and antibiotic agents in the brain (9–11). In the article by Sharma et al. their figure 1 also emphasizes the extracellular space in the brain, which has received a great deal of attention lately in research on the glymphatic and dural lymphatic systems. These have been implicated in neurodegenerative diseases such as Alzheimer's disease or normal pressure hydrocephalus (12). CMD provides a unique method for *in vivo* sampling of this space. The early focus of CMD has been on the small metabolites which can show energy dysfunction in the brain. This is a natural fit for the commercially available and FDA-approved microdialysis catheters, which have an upper molecular weight cutoff of about 20 kDa. There are newer (non-FDA approved) catheters which have a molecular weight cut-off of 100 kDa. These have been used in research protocols to examine proteins such as cytokines, chemokines, and growth factors in the extracellular space, in addition to monitoring small molecules. The interaction of proteins in normal and abnormal situations ranging from TBI to Alzheimer's disease may be explored by analysis of proteins in the glymphatic fluid (13, 14). As most proteins are >20 kDa in size, the growing use of 100-kDa microdialysis catheters has great promise for future studies.

In summary, studies with CMD over the last 40 years have provided a great deal of information about cerebral metabolism, with a focus on glucose metabolism in acute brain injury. Techniques for optimal placement have been worked out by neurosurgeons and neurocritical specialists. One of the main criticisms of CMD is the intermittent nature of sampling (every 30–60 min). Technological advances are forthcoming, and there is great hope that continuous on-line analyzers such as the MD System Loke (M Dialysis) may provide “real-time CMD” (Stovell et al.) (15). With the new interests in the glymphatic and dural lymphatic systems, CMD may grow in value as an important technique for studying the protein components of the extracellular cerebral fluid.

## Author contributions

JC: Conceptualization, Project administration, Supervision, Validation, Writing—original draft, Writing—review and editing. AV: Conceptualization, Project administration, Supervision, Validation, Writing—review and editing. MR: Conceptualization, Project administration, Supervision, Validation, Writing—review and editing. KC: Conceptualization, Project administration, Supervision, Validation, Writing—review and editing.
